# Predict respiratory distress syndrome by umbilical cord blood gas analysis in newborns with reassuring Apgar score

**DOI:** 10.1186/s13052-020-0786-8

**Published:** 2020-02-12

**Authors:** Giuseppe De Bernardo, Rita De Santis, Maurizio Giordano, Desiree Sordino, Giuseppe Buonocore, Serafina Perrone

**Affiliations:** 1 Department of Pediatrics, Neonatology and Neonatal Intensive Care Unit, Buon Consiglio Fatebenefratelli Hospital, Via Manzoni 220, 80123 Napoli, Campania Italy; 2 School of specialization in Pediatrics, Catholic University of the Sacred Heart Faculty of Medicine and Surgery, Rome, Italy; 3 Faculty of Medicine, Federico II University, Naples, Italy; 4 Department of Emergency, NICU A.O.R.N. Santobono-Pausilipon, Naples, Italy; 5 Department of Molecular and Developmental Medicine, University of Siena, Siena, Italy; 6 Department of Medicine and Surgery, University of Parma, Parma, Italy

**Keywords:** NICU, Delivery room, Resuscitation, pH

## Abstract

**Background:**

Neonatal acidaemia at birth can increase neonatal morbidity and mortality and it is predictive of neonatal asphyxia. The umbilical blood gas analysis is a valid tool for the evaluation of neonatal acidaemia. However, umbilical cord blood gas analysis is commonly performed in high-risk situations or in the setting of Apgar scores < 7 at 5 min.

**Methods:**

A retrospective cohort study was conducted from June to December 2018 at the Department of mother’s and child’s health, Poliambulanza Foundation Hospital Institute. Inclusion criteria were: full term newborns with body weight appropriate for gestational age, born by vaginal delivery or caesarean section, reassuring Apgar Score > 7 at 5 min, arterial cord blood gas analysis showing pH < 7.4 or BE <-8 mmol/l or lactate > 6 mmol/l. The aim was to evaluate the predictive role of blood gas analysis for respiratory distress syndrome in newborns with reassuring Apgar Score.

**Results:**

352 full term newborns were enrolled. Umbilical cord blood artery pH showed an association with respiratory distress syndrome (χ^2^(1) = 10,084, OR (95% CI): 3,9 × 10^− 4^(2,9 × 10^− 6^ - 0,048); *p* < 0,05). ROC curve revealed that the cut-off point of pH was 7.12, with a sensibility and specificity of 68 and 63%, respectively.

**Conclusions:**

Umbilical cord blood artery pH < 7.12 at birth is associated to respiratory distress syndrome in newborns. Blood gas analysis is an important instrument to help health care providers during assistance in the delivery room, but also to early identify newborns at high risk for respiratory distress syndrome and better manage the care of these newborns after birth.

## Background

At birth, each newborn is given a score calculated using the Apgar score. This system is a rapid method to assess the clinical status of the newborn at 1 min and 5 min after birth. The score was formulated to have a rapid assessment of the clinical status of the newborn. Therefore, the Apgar score offers the opportunity to report the status of the newborn after birth and the response to resuscitation when performed. The Apgar index is a score obtained from the evaluation of 5 parameters (skin colour, tone, heart rate, crying, respiratory activity) for each parameter a score is assigned by the operator [1]. The incidence of low Apgar scores is inversely related to birth weight and gestational age but only a low score cannot predict morbidity or mortality for each individual newborn [2]. For this reason, the score is a useful tool for the decisions to be taken in the delivery room during the care of the newborn, but it is not a good idea for the assessment of short and long term health status. Differently a tool that can help in assessing the patient’s health status is Blood Gas Analysis (BGA). The BGA allows the evaluation of respiratory exchanges, metabolism and the electrolytic state of the patient [3]. BE is a parameter that evaluates the excess of bases. The reference value is between − 2/+ 2 mmol/l. When this value becomes negative it means that there is a lack of bases and that the patient is in a condition of metabolic acidosis. Lactic acid is produced by cellular metabolism and in hypoxic conditions cells can use less efficient energy production causing excessive production or poor elimination of lactates. pH is the result of the balance between lactates that tend to decrease pH and BE which tend to compensate [4]. Neonatal acidaemia is correlated with an increased risk of admission in neonatal intensive care unit (NICU), hypoxic ischemic encephalopathy, respiratory distress syndrome (RDS) [5, 6], multi-organ disfunction and neonatal exitus [7]. Umbilical cord blood gas analysis is important to evaluate neonatal acidaemia during delivery. Umbilical cord BGA is generally required for Apgar Score < 7^V^ and in newborns with a high risk of asphyxia. Nowadays, there are not clear threshold value of pH, BE and lactate. It is not clear if newborns with moderate acidaemia and Apgar score ≥ 7^V^ must be monitored for development of adverse outcome. Newborns with a good Apgar score have a residual risk of neonatal acidaemia and adverse outcomes [7]. Furthermore, Hermansen at al. described the “acidosis paradox”: newborns without acidaemia at birth might still develop a hypoxic condition. Indeed, in newborns with a normal pH might occur adverse outcomes [8, 9]. Our study analysed BGA to understand if the umbilical blood acidaemia was predictive for RDS in newborns with reassuring Apgar Score > 7 at 5 min. The secondary aim was evaluating if acidaemia in umbilical blood gas analysis was a risk factor for resuscitation in delivery room and admission in NICU.

## Material and methods

### Participants

A retrospective cohort study was conducted from June to December 2018 at the Department of mother’s and child’s health, Poliambulanza Foundation Hospital Institute. The study was conducted in accordance with the Declaration of Helsinki. In this study were enrolled full term newborns with body weight appropriate for gestational age, born by vaginal delivery or instrumental labor or by caesarean section and that showed at blood gas analysis by arterial umbilical cord the following parameters: pH < 7.4, BE <-8 mmol/l, lactate > 6 mmol/l. Preterm newborns were excluded from the study. In delivery room physicians or midwives evaluated Apgar Score at 1 min and 5 min and collected arterial umbilical cord blood within 15 min from delivery. BGA evaluation was a routine practice at birth. Neonatologists were present at all instrumental deliveries and caesarean sections but only at the request of midwives during vaginal deliveries All babies received the same clinical management the environment was strictly controlled, the delivery room temperature did not have to rise less 26 °C [10], while the luminosity was set at 2000 lx and the noises did not get over 45 dB. RDS was recognised as any signs of breathing difficulties in the neonate: Silverman score. Designed by US paediatricians in 1956 it is a clinical evaluation of the state of RDS. It is based on the evaluation of five characteristics to which a score from 0 to 2 can be given: nasal fin finning, thoracic retractions, intercostal recesses, recesses to the jugulum and groaning [5, 6]. Silverman score was performed in newborns with reassuring Apgar Score (> 7) at 5 min after birth.

### Instruments

The following clinical features of the newborns were recorded in a database: gender, gestational age, body weight, Apgar score, pH, BE, lactate, delivery mode, hypoglycaemia at 2 h, hypothermia, admission in NICU, RDS. Nurses that were on duty measured the body weight of the newborn by Eura Mod.AS/1 O.M.I.P. Milano and temperature by Filac™ 3000 AD electronic thermometer. Glycaemia and BGA were measured by ABL90 FLEX (Radiometer). RDS is recognised as any signs of breathing difficulties in the neonate (Silverman score) by trained physicians [5, 6].

### Statistical analysis

A statistician that was aware of the study aims using IBM SPSS Statistics for Windows, v.25, carried out statistical analysis (Armonk, NY: IBM Corp.). Sample size was computed setting α = 0.05, β = 0.05, Odds Ratio = 4.6 [7] and obtaining 352 as sample recruitable. Normal distribution was evaluated by Kolmogorov Smirnov test. Differences of pH value among delivery mode was obtained by one-way ANOVA. Binary logistic regression was executed to evaluate the factors that can be predictive for RDS, access in NICU and neonatal resuscitation. Receiver operating characteristic (ROC) was performed to establish cut off points of the BGA to be predictive of RDS and admission in NICU. Bayes’ theorem analysed the probability that RDS or access in NICU or neonatal reanimation were present in a newborn with acidaemia. Likelihood ratio, sensibility, specificity, positive predictive value (PPV) and negative predictive value (NPV) established if BGA was a good screening tool. Differences were statistically significant with *p* < 0,05.

## Results

**Fig. 1:**
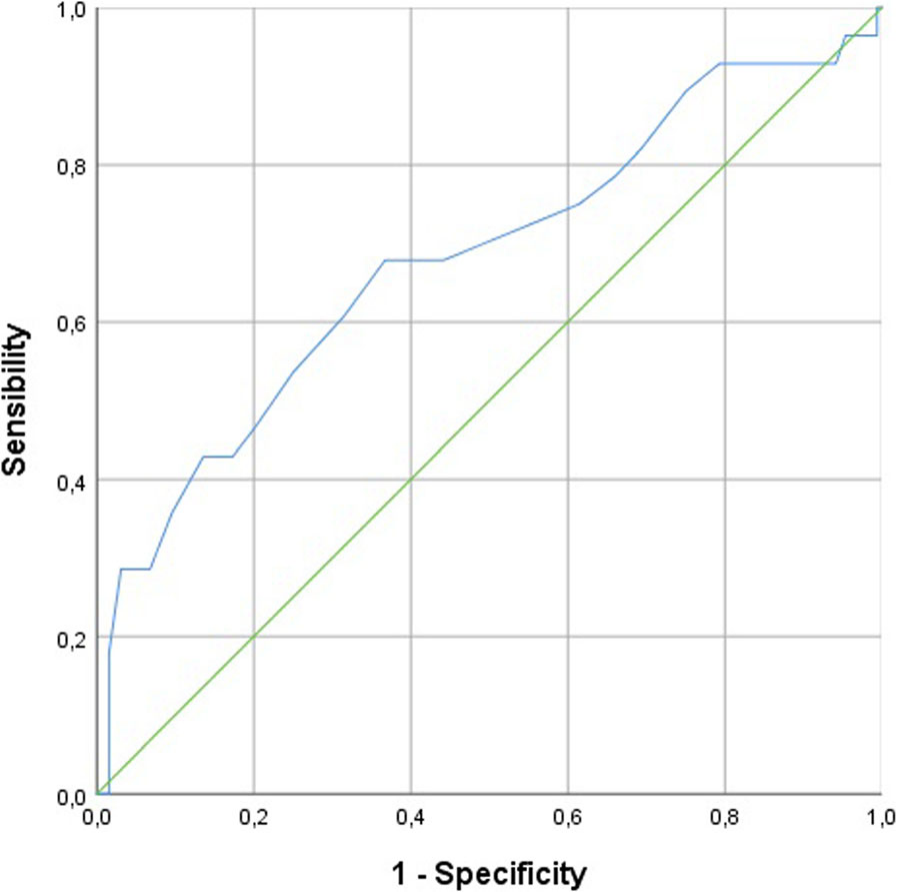
ROC curve for pH and RDS. Blu line represent the trend of sensibility and specificity in function of pH values. Green line is a reference line

**Fig. 2:**
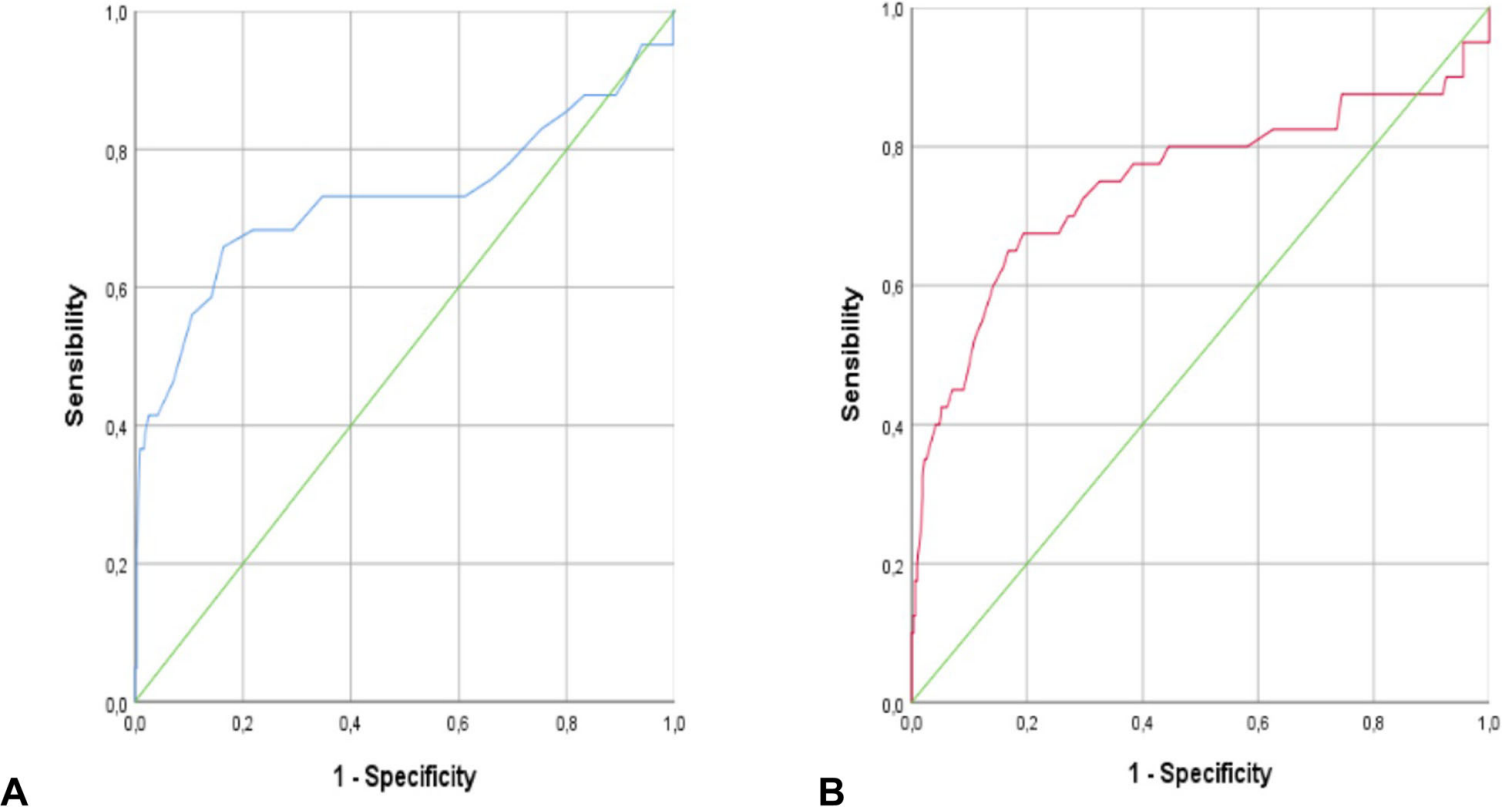
**a** ROC curve for pH and access in NICU. Blu line represent the trend of sensibility and specificity in function of pH values. B, ROC curve for lactate and access in NICU. Red line represents the trend of sensibility and specificity in function of lactate values. **a**, **b** Green line is a reference line

A total of 352 full term newborns were enrolled for the study. Clinical characteristics of population study were reported in Table 1. Newborns born by vaginal delivery or elective caesarean section showed similar pH values (*p* > 0,05). Newborns born by these two modalities of birth presented higher pH values than those born by urgency/emergency caesarean section or instrumental labor (*p* < 0,05). Similar pH values were revealed in newborns born by instrumental labor or emergency/urgency caesarean section (*p* > 0,05). Analysing BGA parameters, pH showed an association with RDS (Table 2). ROC curve revealed that the cut-off point of pH was 7.12 (Fig. 1), with a sensibility, specificity, with PPV and NPV of 68, 63, 14 and 96%, respectively. Hypothermia and hypoglycaemia were also predictive for RDS onset (Table 2). Furthermore, BGA parameters, hypothermia and RDS were predictive for the access in NICU **(**Table 3). ROC curve revealed that only pH and lactate were the best predictive for the access in NICU (*p* < 0,05). The cut-off point of pH was 7.10 with sensibility, specificity, PPV and NPV of 68, 78, 29 and 95% respectively, while the cut-off point of lactate was 8.25 mmol/l with sensibility, specificity, PPV and NPV of 68, 81, 30 and 94% respectively (Fig. 2a-b). pH and BE were predictive of neonatal resuscitation (χ^2^(1) =4749; *p* < 0,05; OR = 0,995 (0,990-0,999). The overall incidence of umbilical artery pH < 7.12 was 33.8% (*N* = 119). Out of these 119 newborns, 14% showed RDS, 24% had access in NICU and 15% received neonatal resuscitation.

**Table 1:** Clinical characteristics of the population study

	(Mean ± SD)
Gender, %	F = 45.7 M = 54.3
Gestational age, wks	39.7 (1.1)
Body Weight, g	3284.7 (394)
Emergency or Urgency caesarean section	8,7%
Elective caesarean section	2,8%
Instrumental labor	24.7%
Vaginal delivery	63.7%
Apgar Score 1 min, median (25^o^-75^o^ percentile)	9 (8–9)
Apgar Score 5 min	10 (9–10)
pH of the newborns born by vaginal delivery	7.15 (0.074)
pH of the newborns born by instrumental labor	7.11 (0.076)
pH of the newborns born by elective caesarean section	7.20 (0.070)
pH of the newborns born by emergency or urgency caesarean section	7,08 (0,066)
pH	7,14 (0,078)
BE, mEq/l	3.4 (9.2)
Lactate, mmol/l	6.7 (2.9)
pO_2_, mmHg	24 (7,2)
pCO_2_, mmHg	52 (9,7)

Data are expressed in Mean ± SD, Median, Percentiles, Percentages as appropriate

**Table 2:** Blood gas analysis parameters in relation to respiratory distress syndrome onset

Predictive Factors	χ-square test	DF	*p*_value	OR (95% CI)
pH	10,084	1	***p*** **< 0,05**	3,9 × 10^−4^(2,9 × 10^−6^ - 0,048)
pH & BE	1787	1	*P* > 0,05	0,974 (0,938-1012)
pH & BE & Lactate	0,577	1	*p* > 0,05	1062 (0,902-1250)
Hypothermia & pH	2804	1	***p*** **< 0,05**	0,368 (0,124-1093)
Hypoglycaemia & pH	19,339	1	*p* > 0,05	0,694 (0,611-0,790

*DF* degree freedom, *OR* odds ratio

**Table 3:** Blood gas analysis parameters in relation to the need to access in NICU

Predictive Factors	χ-square test	DF	*p*_value	OR (95% CI)
pH	30,081	1	***p*** **< 0,05**	4 × 10^−6^(2,94 × 10^−8^-4,95 × 10^−4^)
pH & BE	6883	1	***p*** **< 0,05**	0,956 (0,924-0,989)
pH & BE & Lactate	11,976	1	***p*** **< 0,05**	0,997 (0,995-0,999)
Hypothermia & pH	76,041	1	***p*** **< 0,05**	0,006 (0,001-0,028)
Hypoglycaemia & pH	1063	1	*p* > 0,05	0,465 (0,117-1838)
RDS & pH	52,160	1	***p*** **< 0,05**	0,030 (0,011-0,084)

*BE* base excess, *DF* degree freedom, *OR* odds ratio, *RDS* respiratory distress syndrome

The probability that one of the following conditions occurred in newborns were:

- RDS given a pH < 7.12 (*p* = 0.14, odds = 0.08, positive likelihood ratio = 0,06)
- Access in NICU given a pH < 7.12 (*p* = 0.23, odds = 0.13, positive likelihood ratio = 0,03)
- Neonatal resuscitation given a pH < 7.12 (*p* = 0.15, odds = 0.11, positive likelihood ratio = 0,02)

Acidaemia and RDS occurred in 4.7% of the enrolled newborns, while acidaemia and access in NICU occurred in 8% of enrolled newborns.

## Discussion

Our study was conducted to evaluate the predictive role of pH, BE and lactate in umbilical cord BGA, for respiratory outcome of newborns after birth. Umbilical cord blood gas analysis is important to evaluate the newborn acidaemia during childbirth [7]. Actually, the status of the newborn immediately after birth is evaluated through the Apgar score which represents a rapid tool to assess the clinical status of the newborn at 1 min and 5 min after birth [11]. Apgar score also provides an accepted and convenient method to report response to resuscitation when performed [12]. The score is decided on the evaluation of clinical characteristics, but the score is subjectively assigned. It is also conditioned by maternal sedation or anaesthesia, congenital malformations, gestational age, trauma [13]. The healthy preterm infant without evidence of asphyxia can still receive a low score for immaturity [14, 15] and a low score cannot predict morbidity or mortality for each individual child [2]. It is important to recognize the limits of the Apgar score to be able to use it appropriately. Neonatal acidaemia is related to an increased risk of admission to the neonatal intensive care unit (NICU) due to respiratory difficulties, hypoxic ischemic encephalopathy, multi-organ dysfunction and neonatal exit [7]. However, the performance of arterial blood gas analysis of the umbilical cord is not performed routinely at each delivery. The analysis is required when the Apgar score is < 7 or when the newborn has a high risk of asphyxiation. Newborns who are advised to perform the cord blood gas analysis are those in whom electronic foetal monitoring presents anomalies. In these cases, the execution of the analysis is carried out to assess the presence of acidaemia and therefore the possible hypothermia treatment [16]. In 2010 a meta-analysis by Malin et al. showed that arterial cord pH was significantly associated with neonatal mortality (odds ratio 16.9, 95% confidence interval 9.7 to 29.5, I2 = 0%), hypoxic ischemic encephalopathy (13.8, 6.6 to 28.9, I2 = 0%), haemorrhage intraventricular or periventricular leukomalacia (2.9, 2.1 at 4.1, I2 = 0%) and cerebral palsy (2.3, 1.3 to 4.2, I2 = 0%). However, a universal pH value has not been identified as cut off for an increased risk of long-term adverse outcomes. As far as pH value is concerned, there is a wide heterogeneity with a wide predictive range (0.0–38,169.8). The pH of 7.00 as cut off for negative events did not reach the overall importance with a wide predictive range. The results of a threshold of 7.10 gave an estimate of the similar point but reached a meaning. However, the predictive interval remained wide (0.8–64.3) and went through the line of no effect. For a threshold of 7.20, the odds ratio was lower, with a large predictive range (0.5–40, 6). Only one study examined all three thresholds, one with the strongest association at the threshold 7.00 and the weakest to 7.20. Moreover, the possible correlation between BE and lactates and neonatal morbidity has not been assessed in the studies considered in the meta-analysis [17–24]. In our study the parameter of the BGA that has the greatest predictive capacity for adverse events was pH. ROC curve revealed that the pH break point was 7.12, with a sensitivity e specificity of 68 and 63% respectively. BGA, hypothermia and RDS were predictive for the access in NICU. The pH level that predict to an entry in NICU was 7.10 with a sensitivity of specificity of 68 and 78% respectively, while the lactate cut off was 8.25 mmol/l with a sensitivity and specificity of 68 and 81% respectively. Finally, pH and BE were predictive for neonatal resuscitation. In 2012, Yeh at all reported pH values of 51,519 children born at term. The aim was to examine the relationship between the umbilical cord pH and the severe neonatal outcomes. The absolute risks, the relative risks with confidence intervals of 95% and the numbers necessary for the damage were calculated for different levels of arterial pH. The absolute risk of an adverse neurological outcome was significantly increased below 7.10 and it was lower between 7.26 and 7.30 [25]. It is not clear whether infants with moderate acidaemia and Apgar score ≥ 7^V^ should be monitored for the development of adverse neurological outcome. Sabol et all reported that newborns with a good Apgar the score, have a risk of neonatal acidaemia and adverse outcome [7]. Moreover, Hermansen described the “acidosis paradox”: newborns without acidaemia at birth could still develop a hypoxic condition. In fact, in newborns with normal pH and catastrophic intrapartum events adverse outcome may occur [8]. Our results add to the current literature an in deep knowledge about the role of pH value in cord blood to predict respiratory outcome in the newborns. BGA in cord blood allows the early identification of newborns at high risk for RDS and guide the clinicians to better manage the care of the baby immediately after birth. Delivery mode is also reported as a risk factor for adverse events as RDS [5]. pH is an important tool to help health care providers during assistance in the delivery room but also to direct and predict the path of care of the newborn after birth. In our study 33.8% of enrolled newborns showed acidaemia (pH < 7.12), but only 4.7% showed also RDS, suggesting that umbilical cord BGA could be a good primary screening tool to identify all newborns at risk of RDS. It is plausibly that skin to skin could be performed in these newborns, as this procedure reduces stress and favours the regular transition to extrauterine life. The results of the study suggest a strict clinical evaluation of the newborns at risk of RDS in the first hours of life, comprising continuous evaluation of SpO_2_ and heart rate by pulse oximeter, respiratory rate, sucking, colour skin, temperature and the appearance of any breathing difficulties.

## Conclusions

Arterial umbilical cord BGA seems to be a useful tool to early identify newborns at high risk to develop RDS. Arterial cord blood pH < 7.12 even in presence of reassuring Apgar Score represents a risk factor for RDS onset. Hypothermia and hypoglycaemia at birth are additional risk factors for RDS onset. Furthermore, pH, BE, lactate and hypothermia are predictive for the access in NICU.

## Data Availability

The datasets generated and/or analysed during the current study are not publicly available due privacy reasons but are available from the corresponding author on reasonable request.

## Abbreviations

BE: Base excess
BGA: Blood gas analysis
NICU: Neonatal intensive care unit
NPV: Negative predictive value
PPV: Positive predictive value
RDS: Respiratory distress syndrome
ROC: Receiver operating characteristic
